# Antibiotic resistance management

**DOI:** 10.1093/emph/eou024

**Published:** 2014-10-28

**Authors:** Andrew F. Read, Robert J. Woods

**Affiliations:** ^1^Center for Infectious Disease Dynamics, The Pennsylvania State University, University Park, PA 16802, USA and ^2^Division of Infectious Diseases, University of Michigan, Ann Arbor, MI 48109, USA

## Antibiotic resistance

Antibiotic resistance is ‘bacteria changing in ways that reduce or eliminate the effectiveness of antibiotics’ [1]. These changes are due to bacterial evolution, and threaten the single greatest therapeutic advance in the history of medicine.

Antibiotic resistance genes arose long ago in response to naturally occurring antibiotics. Modern medicine has driven further evolution of some of these genes. Resistance can also arise spontaneously by mutation. In bacteria, genes can be inherited or they can be acquired from non-relatives on mobile genetic elements like plasmids. This horizontal gene transfer (HGT) can occur between very different bacteria.

Antibiotic use drives the evolution of resistance [2]. Resistant bacteria spread by natural selection when antibiotics fail to halt their reproduction while removing their drug-sensitive competitors.

Antibiotic resistance management is an attempt to slow the spread of resistance by the judicious use of antibiotics, an important aim of antibiotic stewardship programs.

## Evolutionary perspectives

One way the evolution of antibiotic resistance can be slowed is by minimizing the strength of natural selection for resistance genes. This means reducing antibiotic use. Don’t treat asymptomatic infections. Use antibiotics only where they can work. 70% of acute bronchitis cases are treated with antibiotics to no effect [3]. When treatment is necessary, use as little as possible. Decrease the need for antibiotic treatment with vaccines, hygiene, and isolation of infected patients. Prevent non-medical uses like growth promotion in farm animals.

Reducing antibiotic use also limits selection for resistance in harmless bacteria that can donate genes to pathogens by HGT, as does targeting pathogens with narrow spectrum antibiotics.

Another way to slow resistance evolution is to prevent pathogens acquiring resistance genes in the first place. In some cases, this can be done with high doses (dead bugs can’t evolve) or combination therapy (acquiring resistance to several drugs at once is unlikely).

Bottom line: antibiotics should be used only when necessary and then, appropriately.

## Future clinical implications

What constitutes appropriate use of antibiotics can be controversial. For instance, dead bugs can’t acquire resistance, so aggressively killing pathogenic bacteria with antibiotics can retard resistance evolution. But aggressive treatment also maximizes the evolutionary advantage of already resistant pathogens or of non-target bacteria that can be a source of resistance genes. Consequently, treatments designed to minimize the rate of resistance acquisition might not be best when resistance is already present [4].

Other major research questions: (i) when do drug cocktails select for multidrug resistance faster than sequential monotherapies?, (ii) does repeated antibiotic use generate a microbiomic reservoir of resistance genes for future pathogens?, (iii) what properties of particular drugs make them more evolution-proof than others?, and (iv) is inappropriate antibiotic use the main evolutionary force undermining drug efficacy—or is it medically appropriate use?
